# Repeated harvest enables efficient production of VSV-GP

**DOI:** 10.3389/fbioe.2024.1505338

**Published:** 2024-12-05

**Authors:** Rebecca Habisch, Peter Neubauer, Jorge Soza-Ried, Eva Puschmann

**Affiliations:** ^1^ Boehringer Ingelheim, Viral Therapeutics Center, Ochsenhausen, Germany; ^2^ Department of Bioprocess Engineering, Institute of Biotechnology, Technische Universität Berlin, Berlin, Germany

**Keywords:** VSV-GP, oncolytic virus production, bioprocess development, repeated-batch, perfusion

## Abstract

Viral products keep gaining importance in multiple therapeutic fields. Considering the scale and production slot limitations, optimizing the outcome of every manufacturing batch is essential to minimize costs and make this therapeutic modality broadly available to patients. Most manufacturing processes for oncolytic viruses currently in clinical studies are based on a batch process. Here, we evaluated the benefits in terms of titer increase of a repeated harvest approach and compared it to the classical batch production process. While no effect on cell density was observed, the cumulated infectious titer following repeated harvest was over 400 times higher than the evaluated batch process yield. This shows that repeated harvests or perfusion have the potential to boost viral yields and should be considered when deciding on a process format for production.

## 1 Introduction

In the last decade, viral products gained importance both as gene therapy vectors and oncolytic therapy. Ongoing trials include a gene therapy for hemophilia A via adeno-associated viruses (Clinicaltrials, 2024) and the use of various oncolytic viruses for lung cancer treatment (Sakhi et al., 2023). Vesicular stomatitis virus (VSV) is widely known for its oncolytic properties. In 2011 wildtype VSV was pseudo typed with the glycoprotein (GP) of lymphocytic choriomeningitis virus (LCMV), eradicating previous issues of neurotoxicity (Muik et al., 2011). Since then, the resulting VSV-GP has shown potential for various applications. It was first proven to be a potent viral vaccine vector (Tober et al., 2014), shortly followed by first studies demonstrating its efficacy as cancer therapy (Dold et al., 2016; Kimpel et al., 2018). In 2022 the first clinical trial including VSV-GP started, investigating its efficacy against advanced and refractory solid tumors both as monotherapy or in combination with an immune checkpoint inhibitor (Porosnicu et al., 2022).

Production of VSV and VSV-GP is frequently performed in human embryonic kidney (HEK) cells. The HEK293 cell line was transformed using sheared DNA of human adenovirus (Graham et al., 1977). HEK293 cells can grow in suspension culture, thus providing well-known benefits regarding scalability, and culture handling. Nevertheless, with increasing demand and limited production capacities, the development of reproducible and robust processes resulting in high volumetric virus yields is essential. While the generation of suspension cell cultures has been critical for increasing viral titers, further improvements can be obtained by additives such as caffeine (Ellis et al., 2011), sodium butyrate (Olsen and Sechelski, 1995), potassium chloride or sodium chloride (Yu et al., 2021). Approaches focused on increasing viral titers by improvements in the manufacturing process include the implementation of fed-batch or perfusion. A fed-batch- process ensures the supply of required nutrients via specifically adjusted feed-media. Recently, a fed-batch process has been combined with a mathematical model to further optimize the outcome of baculovirus production (Sharma et al., 2023). Perfusion provides the additional benefit of removing potentially inhibitory host cell metabolites and, depending on the chosen filter, offers a possibility of continuous harvesting. Depending on the stability of a virus at cell culture conditions, perfusion with continuous harvest might be combined with subsequent storage of material to increase the total virus yield. In general, perfusion provides a consistent cellular environment, i.e., by supplying higher cell densities with feed media adjusted to the requirements of distinct process phases. Perfusion faces various challenges for its successful implementation. These include complex process controls and a lack of suitable scale-down models (Mayrhofer and Kunert, 2019). While several studies have already successfully addressed some of the obstacles, such as filter fouling by the use of acoustic settlers in perfusion processes with continuous harvest, a major task still remains to be solved with respect to its implementation for manufacturing at a large scale (Gränicher et al., 2021). There is a need for continuous supply of one or more feed media and a collection of spent medium, requiring additional containers in proximity of the bioreactor. As a result, many production sites established for batch or fed-batch production processes are not fit to accommodate these demands. Furthermore, the addition of harvest additives like NaCl cannot be easily applied in the perfusion set up and poses a specific challenge. An intermediate pulse-based addition of the salt and thus, the introduction of further process steps, would make the process even more complex.

In this study we evaluated the implementation of a perfusion process for the production of a recombinant VSV-GP carrying a cargo immunomodulating protein. For this, repeated batch harvests were performed and compared to a batch process. With the aim of including approaches representing continuous harvest and product retention, respectively, one approach included NaCl treatment before harvests to enhance virus recovery as described previously (Gautam et al., 2022), while another was performed without intermittent NaCl treatments. While this NaCl treatment doubled absolute virus harvest, the main effect was found to originate in media exchange, which caused a 400-fold increase in virus yield compared to the maximum of the batch control. Altogether, these data demonstrate that media exchange has the capacity to intensely boost virus yield in comparison to batch processes performed in unoptimized media. They further suggest that the majority of virus is produced within a short timeframe, although further investigation and closer monitoring is required to be certain.

## 2 Materials and methods

### 2.1 Cell culture and production of recombinant VSV-GP using repeated harvest

HEK293 cells (Thermo Fisher Scientific) were cultured in a chemically defined, serum-free in-house media at 37°C with 5% CO_2_ at 120 rpm. For virus production, HEK293 cells were seeded at 7.5E + 05 cells/mL and kept in the culture conditions described for 96 h in a volume of 25 mL in shake flasks (Corning, #431143). 80% media exchange for titer optimization (Elahi et al., 2019) was then performed via centrifugation at 180 × g, 5 min and exchange of supernatant. Following media exchange, cells were infected with recombinant VSV-GP at an MOI of 0.0005.

At 36, 44 and 52 h post infection (hpi) intermediate harvests were performed. 80% of the culture volume was harvested after centrifugation at 180 × g for 5 min and replaced with fresh medium. A total of three conditions were compared to a batch process. The first condition included treatment of cultures with NaCl before each harvest to increase virus recovery as described previously (Gautam et al., 2022). In the second condition harvests were performed without NaCl treatment. For full evaluation of process formats the third condition combined NaCl-free intermediate harvests with NaCl treatment at only the final harvest. For this, samples were taken from the NaCl free cultures of condition two before the final harvest and treated with NaCl as described for condition one. The batch process (control) was kept in culture without media exchanges and sampled at the same time points as harvests were performed in described test conditions.

### 2.2 Sample collection and treatment

**FIGURE 1:**
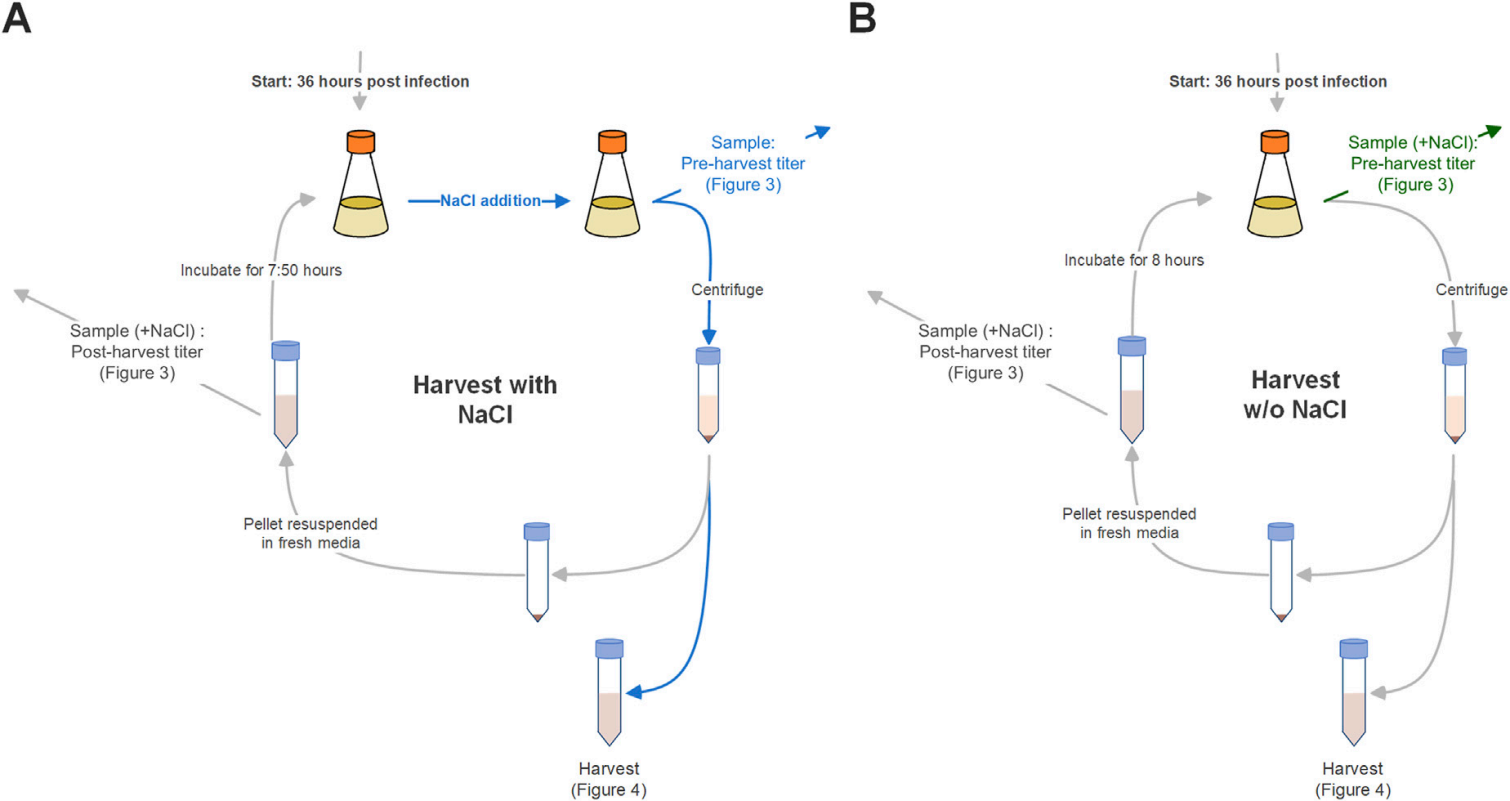
Scheme of repeated harvest setup with **(A)** and without **(B)** NaCl treatment of cultures prior to harvest. Differences in handling are highlighted by blue and green arrows. Blue: Presence of NaCl in the culture and samples. Green: Addition of NaCl to samples of untreated cultures for comparability reasons.

Samples (1 mL) were collected from cultures and harvest fractions, which include all intermediate harvests and the final harvest. Cultures were sampled before every harvest to trace virus production and cell density, and after intermediate harvests to investigate the effect of the intermediate harvest on the respective viral titer in culture. To ensure comparability between the viral titers measured in NaCl-treated and untreated cultures, the samples collected from untreated cultures were subsequently treated with NaCl to have all virus particles released in that sample (see Figure 1, and differences in handling highlighted by blue and green arrows). Samples were centrifuged at 1,000 × g for 5 min, and the supernatant was subsequently aliquoted and stored at −80°C.

### 2.3 Cell count and viability

Cell counts and viability were analyzed from samples taken before harvest steps using a NucleoCounter NC-202 (Chemotec).

### 2.4 Infectious titer analysis via TCID50 assay and calculation of cumulated titer

Samples taken from shake flask and harvest fractions were tested for recombinant VSV-GP infectious titer via TCID_50_ measurement as described previously (Hochdorfer et al., 2022). Briefly, BHK-21 cells were cultured in GMEM -BHK-21 media supplemented with 10% fetal bovine serum and 5% tryptose phosphate broth. They were seeded at 10^4^ cells/well in 96 well plates and infected after 24 ± 4 h 72 ± 4 h post infection wells showing cytopathic effect (CPE) were identified and the infectious titer was calculated using the Spearmen-Kärber method (Spearman, 1908; Kärber, 1931). The cumulated TCID50 was then determined by calculating the absolute TCID50 (titer in harvest multiplied by the harvest volume) for every timepoint and by subsequently adding them together.

### 2.5 Genomic titer analysis via RT-PCR

Viral RNA was extracted using the MagMax Viral/Pathogen Kit (Applied Biosystems, #A42352) in combination with the King Fisher Flex Purification System (Thermo Scientific). Genomic copies were quantified using the TaqMan Fast Virus 1-Step Master Mix (Applied Biosystems, #4444434) and the QuantStudio 6 Flex plate reader.

## 3 Results and discussion

The production yield of recombinant VSV-GP obtained in repeated harvest approaches was compared to the one of a batch process. In addition, it was evaluated whether NaCl treatment before harvests has an impact on the virus yield. Harvests were performed 36, 44, 52 and 60 h post infection.

### 3.1 Cell culture parameters are not affected by repeated harvests

**FIGURE 2:**
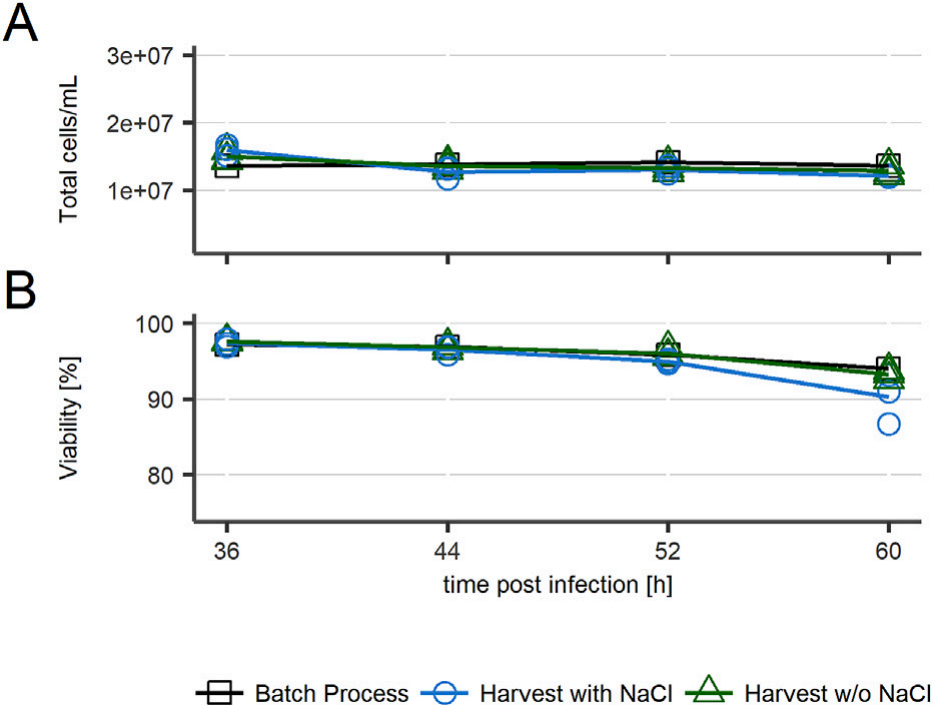
Cell culture parameters for recombinant VSV-GP production in repeated batch harvest mode in HEK293 cells. **(A)** Total cell count/mL **(B)** Viability. n = 3 (biological replicates).

First, the effect of multiple harvests on cell culture was analyzed by measuring the total number of cells and their viability at every intermediate harvest and at the end of the harvest process. As displayed in Figure 2, a slight decrease in viability was observed in the last sample of cultures treated with NaCl before intermediate harvests. In general, total cell count and viability remained similar between all conditions.

### 3.2 VSV-GP titers in culture recovers and increase after intermediate harvests

**FIGURE 3:**
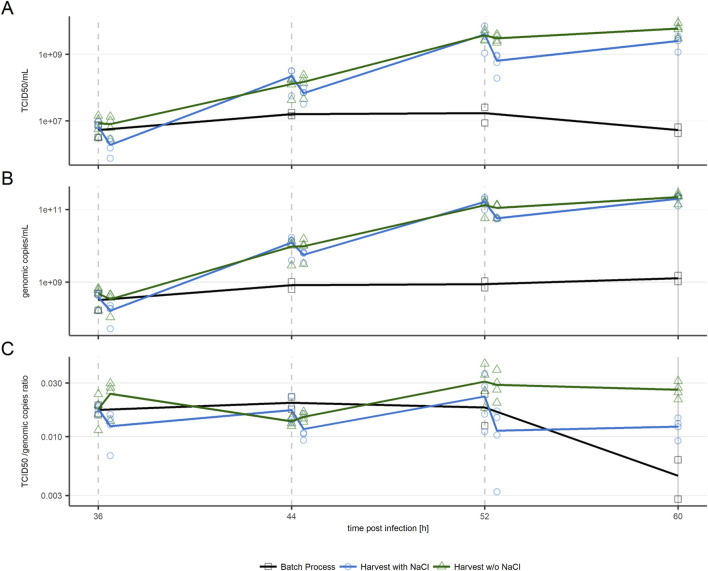
VSV-GP titers in cultures harvested with NaCl treatment before intermediate harvests (blue circles) and without NaCl treatment (green triangles) (n = 4) in comparison to a batch process (black squares, n = 2). **(A)** Infectious particle titer **(B)** Genomic titer **(C)** TCID50/genomic copies ratio. Slashed lines indicate intermediate harvests, the solid line indicates the process end. n = 4 (biological replicates). Respective data is provided in Supplementary Table S1.

Infectious viral titers were tracked by sampling cultures before every harvest step and after addition of fresh media, as displayed in Figure 3. Repeated harvest caused viral titers to reach maxima of over 230 times higher than observed in the batch process. Titers in cultures with intermediate harvests increased until 52 hpi when these intermediate harvests included NaCl treatment (blue line), and until 60 hpi when kept NaCl-free (*p* < 0.05). The batch process contrarily was in, or entered, the plateau phase around 36 hpi. In all evaluated conditions the genomic titer (Figure 3B) showed a similar trend as the infectious titer. The ratio of infectious to genomic titer followed no visible trend for repeated harvest conditions, stating that ratio of infectious to total virus particles was unaffected by the used harvest approach or repeated harvest itself (Figure 3C).

As expected based on previous studies (Gautam et al., 2022), treatment with NaCl to increase virus recovery in harvests resulted in a significant (*p* < 0.05) drop of infectious and genomic titers in respective cultures (Figures 3A, B). Interestingly, these titer drops are unimportant with respect to the increase of virus titer following the first two intermediate harvests. At 44 hpi and 52 hpi, viral titers have increased over 15-fold in comparison to the titer measured in culture before the respective previous harvest. After the third harvest step at 52 hpi, virus production slowed down. Less new virus particles were produced, and the amount of virus removed by intermediate harvest with NaCl treatment was not fully replaced, as shown by the final harvest titers being lower than the ones obtained at 52 hpi. In cultures harvested without NaCl, no significant amount of virus is removed from cultures during the individual harvest steps. Therefore, all produced virus particles accumulated, causing the maximum titer to be reached at the final harvest at 60 hpi.

The increased titer observed in repeated harvest approaches suggests two possible scenarios; the plateau in titer seen in the batch process is due to either media depletion of required nutrients to produce further virus particles, or cell’s virus production/infection are inhibited by spent media.

### 3.3 VSV-GP titers in harvest fractions and cumulative harvest show majority of product in final harvest

**FIGURE 4:**
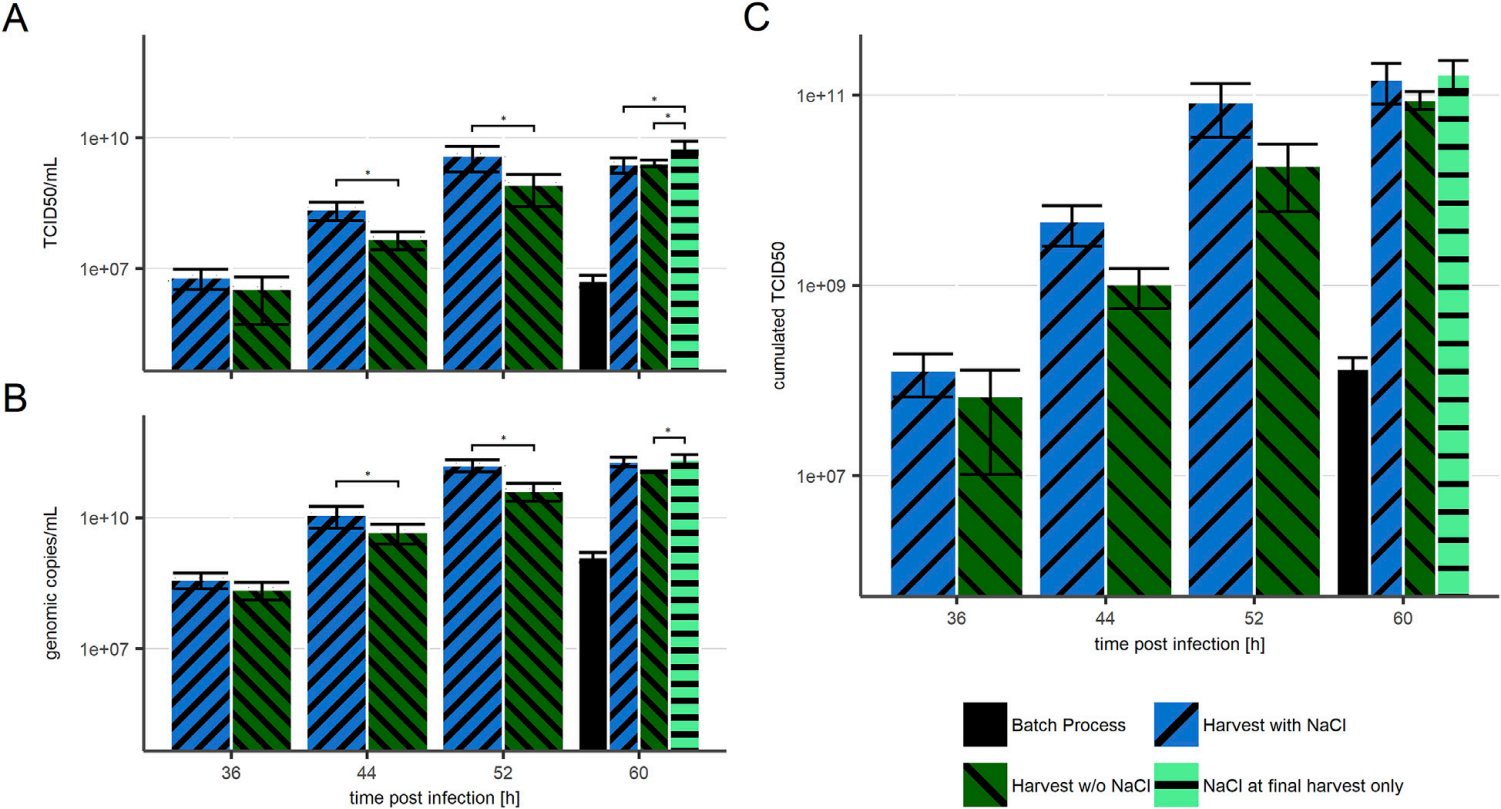
VSV-GP titers in harvest fractions with NaCl treatment before harvests (blue, “/” striped), without NaCl treatment (green, “\” striped), and with NaCl treatment only before the final harvest (light green, “-” striped) in comparison to a batch process (black, solid). **(A)** Infectious particle titer **(B)** Genomic titer **(C)** calculated cumulated infectious titer. n = 4 (biological replicates). Significance has been calculated for differences between measured values in **(A)** and **(B)**. Significance of *p* < 0.05 are indicated by an Asterix (*). The presented data is provided in Supplementary Table S2.

In line with the above, repeated harvest boosted both infectious (Figure 4A) and viral genomic harvest titer (Figure 4B). The highest titers observed in a single (intermediate) harvest fraction were over 150 times higher in all repeated harvest approaches than in the batch process. In cultures harvested with NaCl (blue bars) the harvested titer was equal to the titer measured in the respective culture, while in cultures harvested without NaCl (dark green) a significant difference (*p* < 0.05) was observed between viral titers in cultures and in the harvest. As the viral titers measured at 60 hpi after NaCl-free intermediate harvests were the overall highest titers seen in culture, an additional final harvest including NaCl treatment was performed (light green). When compared to the NaCl-free harvest, this single NaCl step resulted in more than double the amount of infectious virus, which would represent a more than 400 times increase compared to the evaluated batch process.

Similarly, the highest cumulated infectious titers (Figure 4C) were received by using NaCl either for all, or at least for the final harvest at 60 hpi. These two options resulted in similar cumulated infectious titer at the end of the process, which was on average 400 times higher than the virus yield from the batch process (52 hpi). This further indicates that NaCl treatment before intermediate harvests has no influence on the positive effect of media exchange. It merely determines if the majority of infectious viruses is harvested in one culture volume during the final harvest or distributed over multiple harvest volumes.

Considering potential downstream activities, it may be beneficial to keep most produced viruses in a relatively small volume. Thus, the superior condition based on the here presented results would consist of regular media exchanges or perfusion with product retention, and a final harvest including NaCl treatment. Considering the retardation of virus production towards the end of the process, the final harvest would be proposed between 52 and 60 hpi.

Altogether, the demonstrated effect of media exchange boosts the low titer from the batch process by a factor of over 400. Similar titers have been achieved by others for related viruses (Elahi et al., 2019). However, in previous studies the main benefit of perfusion for VSV particle production seems to be based on increased cell density (Göbel et al., 2024). In contrast, the media exchange performed in this study had no effect on the number of cells or another parameter associated to their culture (Figure 2). This suggests either the removal of inhibitors of infection or virus production, or replacement of depleted nutrients by the media exchange.

## 4 Conclusion

The presented study compares repeated harvest approaches with a batch process for the production of recombinant VSV-GP. Here we demonstrated that low viral titers can be boosted by repeated media exchanges via replacement of 80% of intermediate harvest volumes with fresh media. In comparison to the batch process, the total yield of infectious virus was strongly increased, however in processes where media and seed virus have been optimized, effectiveness of media exchange might be lower. The maximum harvest of infectious virus is achieved with NaCl treatment at only the final harvest step, which in this study was at 60 hpi. In the respective approach, all intermediate harvests taken together contained less than 11% of the cumulated virus harvest. Considering the TCID50 assay has a geometric coefficient of variance (GCV) of up to 21% (Hochdorfer et al., 2022), this amount is negligible regarding total harvest. Thus, titer collection from intermediate harvest does not increase the final harvest titer sufficiently to justify the implementation of such a complex process manufacturing step. The highest increase in viral titers was observed between 44 and 52 hpi. After 52 hpi, viral particle production slowed down, but did not stop entirely. This suggests the majority of virus is produced at some point around 52 hpi; based on unpublished data regarding the cycle time for recombinant VSV-GP, we assume an 8-h production window. In summary, we showed that media exchanges can boost previously low viral titers in a batch process by more than 2 orders of magnitude. While media exchange was highly beneficial, semi-continuous harvest brought no benefit. Lastly, the effect of NaCl treatment prior to harvest efficiently improved virus particle release and doubled total virus yield in comparison to NaCl-free final harvest.

## Data Availability

The raw data supporting the conclusions of this article will be made available by the authors, without undue reservation.
