# Supplementary material for: Repeated harvest enables efficient production of VSV-GP
Source: Front Bioeng Biotechnol. 2024 Dec 5;12:1505338. doi: 10.3389/fbioe.2024.1505338 (PMC11656157; doi:10.3389/fbioe.2024.1505338)
Supplement: Supplementary file 1 [file DataSheet1.PDF]

Supplementary Table 1: Collected data of viral titers in culture (displayed in Figure 3). Time points ending in “0.5” indicate a post-harvest sample.

Condition	Colour in figures	hpi	Replicate	TCID50/mL	genomic copies/mL
control	black	36	1	7,50E+06	4,80E+08
with NaCl	blue	36	1	9,31E+06	5,08E+08
no NaCl	dark green	36	1	5,62E+06	4,92E+08
control	black	36	2	3,16E+06	1,66E+08
with NaCl	blue	36	2	6,98E+06	4,43E+08
no NaCl	dark green	36	2	1,43E+07	5,99E+08
with NaCl	blue	36	3	7,50E+06	4,28E+08
no NaCl	dark green	36	3	1,07E+07	6,74E+08
with NaCl	blue	36,5	1	2,37E+06	1,87E+08
no NaCl	dark green	36,5	1	1,00E+07	3,66E+08
with NaCl	blue	36,5	2	1,54E+06	2,28E+08
no NaCl	dark green	36,5	2	1,33E+07	4,47E+08
with NaCl	blue	36,5	3	2,94E+06	1,87E+08
no NaCl	dark green	36,5	3	6,04E+06	4,39E+08
with NaCl	blue	36,5	4	7,50E+05	5,19E+07
no NaCl	dark green	36,5	4	2,74E+06	1,08E+08
control	black	44	1	1,43E+07	6,36E+08
with NaCl	blue	44	1	3,16E+08	1,70E+10
no NaCl	dark green	44	1	1,65E+08	1,14E+10
control	black	44	2	1,78E+07	1,01E+09
with NaCl	blue	44	2	3,16E+08	1,38E+10
no NaCl	dark green	44	2	1,24E+08	1,00E+10
with NaCl	blue	44	3	1,91E+08	1,42E+10
no NaCl	dark green	44	3	1,78E+08	1,36E+10
with NaCl	blue	44	4	5,62E+07	3,99E+09
no NaCl	dark green	44	4	4,22E+07	2,85E+09
with NaCl	blue	44,5	1	6,98E+07	6,59E+09
no NaCl	dark green	44,5	1	1,33E+08	9,83E+09
with NaCl	blue	44,5	2	1,00E+08	6,23E+09
no NaCl	dark green	44,5	2	1,78E+08	1,07E+10
with NaCl	blue	44,5	3	6,98E+07	6,46E+09
no NaCl	dark green	44,5	3	2,37E+08	1,53E+10
with NaCl	blue	44,5	4	3,16E+07	3,40E+09
no NaCl	dark green	44,5	4	4,53E+07	3,13E+09
control	black	52	1	8,66E+06	6,92E+08
with NaCl	blue	52	1	3,40E+09	2,14E+11
no NaCl	dark green	52	1	5,23E+09	1,47E+11
control	black	52	2	2,55E+07	1,07E+09
with NaCl	blue	52	2	6,98E+09	1,92E+11
no NaCl	dark green	52	2	2,74E+09	1,52E+11
with NaCl	blue	52	3	4,22E+09	1,51E+11

no NaCl	dark green	52	3	4,22E+09	1,65E+11
with NaCl	blue	52	4	1,07E+09	9,71E+10
no NaCl	dark green	52	4	2,55E+09	5,72E+10
with NaCl	blue	52,5	1	8,66E+08	5,85E+10
no NaCl	dark green	52,5	1	3,92E+09	1,31E+11
with NaCl	blue	52,5	2	9,31E+08	5,50E+10
no NaCl	dark green	52,5	2	3,40E+09	1,29E+11
with NaCl	blue	52,5	3	5,62E+08	5,46E+10
no NaCl	dark green	52,5	3	2,55E+09	1,27E+11
with NaCl	blue	52,5	4	1,91E+08	5,93E+10
no NaCl	dark green	52,5	4	2,21E+09	5,62E+10
control	black	60	1	4,22E+06	1,52E+09
with NaCl	blue	60	1	3,40E+09	2,32E+11
no NaCl	dark green	60	1	8,66E+09	2,77E+11
control	black	60	2	6,49E+06	1,04E+09
with NaCl	blue	60	2	2,94E+09	2,25E+11
no NaCl	dark green	60	2	6,49E+09	2,38E+11
with NaCl	blue	60	3	2,55E+09	2,08E+11
no NaCl	dark green	60	3	5,62E+09	2,27E+11
with NaCl	blue	60	4	1,15E+09	1,26E+11
no NaCl	dark green	60	4	2,94E+09	1,37E+11

Supplementary Table 2: Collected data of viral titers in harvest fractions (displayed in Figure 4).

Condition	Colour in figures	hpi	Replicate	TCID50/mL	genomic copies/mL
control	black	60	2	6,49E+06	1,04E+09
control	black	60	1	4,22E+06	1,52E+09
with NaCl	blue	36	4	2,05E+06	1,65E+08
with NaCl	blue	44	4	1,00E+08	2,87E+09
with NaCl	blue	52	4	1,33E+09	9,89E+10
with NaCl	blue	60	4	1,15E+09	1,26E+11
with NaCl	blue	36	1	9,31E+06	5,08E+08
with NaCl	blue	44	1	3,16E+08	1,70E+10
with NaCl	blue	52	1	3,40E+09	2,14E+11
with NaCl	blue	60	1	3,40E+09	2,32E+11
with NaCl	blue	36	2	6,98E+06	4,43E+08
with NaCl	blue	44	2	3,16E+08	1,38E+10
with NaCl	blue	52	2	6,98E+09	1,92E+11
with NaCl	blue	60	2	2,94E+09	2,25E+11
with NaCl	blue	36	3	7,50E+06	4,28E+08
with NaCl	blue	44	3	1,91E+08	1,42E+10
with NaCl	blue	52	3	4,22E+09	1,51E+11
with NaCl	blue	60	3	2,55E+09	2,08E+11
no NaCl	dark green	36	4	4,53E+05	8,49E+07
no NaCl	dark green	44	4	1,65E+07	1,39E+09

no NaCl	dark green	52	4	1,33E+08	1,54E+10
no NaCl	dark green	36	1	2,55E+06	2,58E+08
no NaCl	dark green	44	1	6,04E+07	5,60E+09
no NaCl	dark green	52	1	6,98E+08	4,62E+10
no NaCl	dark green	60	1	2,55E+09	1,14E+11
no NaCl	dark green	36	2	7,50E+06	2,87E+08
no NaCl	dark green	44	2	5,62E+07	6,23E+09
no NaCl	dark green	52	2	1,07E+09	5,21E+10
no NaCl	dark green	60	2	2,21E+09	1,08E+11
no NaCl	dark green	36	3	3,40E+06	2,97E+08
no NaCl	dark green	44	3	6,04E+07	6,18E+09
no NaCl	dark green	52	3	1,54E+09	5,69E+10
no NaCl	dark green	60	3	3,16E+09	1,18E+11
no NaCl except final harvest	light green	60	4	2,94E+09	1,37E+11
no NaCl except final harvest	light green	60	1	8,66E+09	2,77E+11
no NaCl except final harvest	light green	60	2	6,49E+09	2,38E+11
no NaCl except final harvest	light green	60	3	5,62E+09	2,27E+11
